# Revisiting Proteus 2.0: Two Decades of Pioneering Lectin Crystallography at BioMol-Lab in Northeast Brazil

**DOI:** 10.1021/acsomega.5c03011

**Published:** 2025-06-12

**Authors:** Benildo S. Cavada, Kyria S. Nascimento, Vinicius J. S. Osterne

**Affiliations:** Department of Biochemistry and Molecular Biology, Federal University of Ceara, Fortaleza 60020-181, Ceará, Brazil

## Abstract

This narrative review reports two decades of pioneering X-ray crystallography research on plant lectins at the BioMol-Lab (Northeast, Brazil). Inspired by the shape-shifting Greek god Proteus, our work has consistently highlighted the structural plasticity and functional diversity of these carbohydrate-binding proteins. From modest beginnings, relying heavily on international collaborations, BioMol-Lab established the first protein crystallization laboratory outside of São Paulo, Brazil, becoming a regional and national leader in structural biology. We detail the evolution of our research, from early successes in solving structures of ConA-like lectins and the novel Jacalin-related lectin from Parkia platycephalla, to our current integration of high-throughput crystallography, advanced synchrotron techniques (utilizing Brazil’s LNLS and Sirius facilities), and computational methods (Molecular modeling, molecular docking, and molecular dynamics simulations). Key findings include the identification of noncanonical binding sites (e.g., for α-aminobutyric acid and indole-3-acetic acid), the elucidation of pH-dependent oligomerization mechanisms, and attempts to define the structural basis for the diverse biological activities of plant lectins. The combination of structural determination with predictive bioinformatics is also discussed. This review emphasizes the importance of small structural variations in dictating the outcomes of these proteins in various biological activities and showcases the power of combining structural biology with Brazilian biodiversity to generate novel insights into plant lectin biology and biotechnological potential.

## Graphical abstract

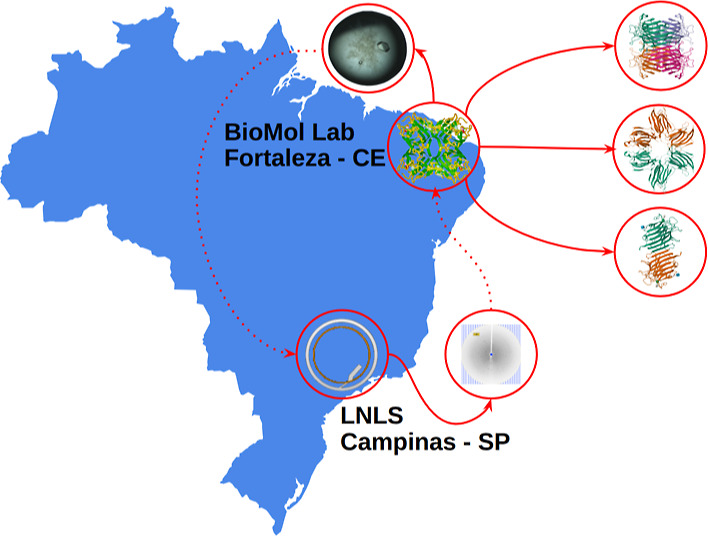

## 1 Introduction

### 1.1 The Allure of Lectins

The ever-growing body of literature on plant lectins, a subject consistently fascinating to us, highlights their potential across various fields. Lectins are nonimmune proteins that bind carbohydrate structures through a specific binding site known as the carbohydrate-recognition domain (CRD) in their structures. This binding is both specific and reversible, and does not alter the structure of the ligand.^1^ However, despite their high specificity, the lectin-carbohydrate interactions can be characterized by a relatively low affinity, with dissociation constants (*K*_d_) in the millimolar (mM) to micromolar (μM) range. This weak binding happens due to the nature and the number of interactions, which are primarily noncovalent and include hydrogen bonds, van der Waals forces, and hydrophobic interactions.^2,3^ For example, lectin-carbohydrate interactions often exhibit values around 0.1–1 mM for monosaccharides. In contrast, antibody–antigen interactions are significantly stronger, with values typically in the nanomolar (nM) to picomolar (pM) range, reflecting much higher affinity due to the higher structural complementarity and multiple noncovalent bonds involved.^4,5^ This low affinity can be compensated through multivalency, a phenomenon where multiple CRDs bind simultaneously with multiple carbohydrates on a ligand. This enhances binding strength and allows for a stable binding despite the weak individual affinities, and affinity increases reaching numbers such as 10,000 fold.^6^

The function of lectins in plants is still a blooming area of research, but some strong conclusions have been made over the years. During the early days of our engagement in this area, in the 1970s, the consensus was directed toward their roles as storage proteins. This role was suggested considering the high concentration (up to 40%) of lectins in seeds and other storage organs.^7,8^ This was a hypothesis with which we did not fully agree, as we firmly believed that plants would not produce such interesting proteins for such a basic application, especially when vicilins, such as Canavalin, Phaseolin and similar proteins are already immensely present in seeds.^9,10^ Vigorous discussions with supervisors and a strong background in agronomic sciences and plant physiology led us to stand against this hypothesis and, in fact, in two of our works, we verified that lectins from Canavalia brasiliensis (ConBr) and Pisum arvense (PAL) displayed remarkable resilience during seed germination, maintaining their structural integrity and functional activity even as the well-known storage proteins were rapidly mobilized and degraded. This was evident from the displayed hemagglutination activity and unchanged electrophoretic profiles of these lectins during extended periods of germination. In P. arvense, for instance, lectins resisted proteolytic breakdown and remained functionally active until cotyledons were fully depleted of reserves. This behavior contrasts with high-molecular-weight storage proteins, which undergo systematic proteolysis to supply amino acids for seedling development. Similarly, ConBr showed comparable stability under both light and dark conditions during germination. These results corroborate our views and further question the simplistic view of lectins as storage proteins and instead point to their potential involvement in plant defense mechanisms and physiological regulation during early development stages.^11,12^

An important review to the field was published many years ago by Sharon (1977). It introduced lectins as fundamental tools for studying cell surface dynamics. Sharon emphasized that lectins are not merely plant proteins, but molecular probes that selectively bind to sugars on cell surfaces. Although it is common now to think of lectins in this way, at the time, this came as a paradigm shift to the field. This property allows lectins to mediate cell–cell interactions and participate in processes including cell growth, differentiation, defense responses among others. The review also introduced that the specificity of lectins can be used for the recognition of carbohydrate moieties on glycoconjugates, kickstarting studies on their use to distinguish between normal and malignant cells. It also highlighted their agglutination capacity, facilitating the mapping of cell surface sugars, blood typing and study of cell glycosylation changes during biological processes.^13^

Our initial suspicions regarding functions beyond storage were further proved right when new research on the roles of plant lectins in defense against pathogens and herbivores piled up, this effect being caused by their antinutritional effect on insects and animals, resulting from the binding with glycoconjugates on the intestines of the attacker, negatively affecting nutrient absorption.^1,14^ We found that the suggestion of this role for plant lectins to be particularly strong, but also saw other suggestions pop up over the years. For example, the participation of legume lectin in the symbiosis process with rhizobia includes a beautiful network of protein-bacteria interactions involving chitooligosaccharides in the bacterial surface and results in increased colonization of rhizobia.^15^ Works from our group and others are also revealing the capacity of some legume lectins to interact with hydrophobic compounds in regions of the protein other than the CRD. This discovery allowed for the suggestion of a role in the transport and regulation of hydrophobic compounds in planta, including plant growth hormones.^16,17^ We also watched closely the discovery of stress-inducible lectins, produced in very low concentrations in normal plant conditions, but significantly overexpressed in stress-conditions. These lectins have clear roles in biotic and abiotic stress responses.^18,19^

A large interest within our laboratory and the lectin field in general is the biological activities and biotechnological potential of plant lectins. It is undeniable that lectins can be applied in several areas and have the potential to become important biotechnological products. Lectins are majorly tested as tools in biomedicine, agriculture and glycobiology. Our own group has its fair share of contributions to this field, and we have been able to watch a boom of new applications and potential for lectins within biotechnology. Our publication list reveals applications of several lectins, but this is something that has been extensively discussed in other reviews in the area.^20,21^

This initial overview indicates the multifaceted nature of lectins, hinting at the complexity that structural studies would later help to unravel, forming the core of BioMol-Lab’s crystallography division research journey.

### 1.2 Lectin Crystallography

It is evident to us that, historically, the most important technique to study the structure of lectins is X-ray crystallography. Crystallography and structure solving have become an indispensable tool in understanding the molecular basis of carbohydrate recognition and overall 3D structure of the proteins. The history of lectin crystallography is a trajectory of scientific discovery, from the identification of lectins in plants by Stillmark in 1888 ^22^ to the atomic-level resolution of complex lectin-glycan interactions in recent decades. Early breakthroughs, such as the structural solving of concanavalin A (ConA) in 1972 by Hardman and Ainsworth,^23^ provided much information into the lectin structure and set the stage for studying many other lectins across species. These first studies were extremely important for the field because they offered the first insights into the CRDs found in the structure of all lectins and are responsible for the binding capacity.^24^ Reflecting on this, it is impressive how early structural insights obtained with limited technology, shaped everything that came later. The structural solving of ConA demonstrated the power of X-ray crystallography in understanding the structural basis of carbohydrate-binding, a subject that remains quite important to the field nowadays.

As the field matured, the appearance of cocrystallization techniques in the 1990s became central, allowing lectinologists to capture lectins complexed with their specific carbohydrates.^25^ These studies not only confirmed the principles of carbohydrate-binding by lectins, which involve hydrogen bond networks, CH-π stacking interactions, hydrophobic and van der Waals interactions, but also provided the capacity to expand knowledge to different contexts and glycans.^26^ For example, the galectin’s ability to bind β-galactosides and their role in modulating immune responses were elucidated through such complexes.^27^ Reflecting on this progression, it is incredible how these technical innovations addressed questions that were once speculative, enabling direct visualization of lectin-ligand interactions.

A lot has been learned about plant lectin binding as well, for example, the molecular basis for legume lectin binding that could be summarized into a structurally conserved β-sandwich fold that supports a carbohydrate-recognition domain (CRD) with specificity-driven variability. Despite the overall similarity in tertiary structure among legume lectins, variations in CRD loop lengths and amino acid compositions contribute to their diverse carbohydrate-binding specificities. This diversity allows legume lectins to be categorized into distinct specificity groups, including mannose/glucose-specific, *N*-acetyl-d-galactosamine/galactose-specific, *N*-acetyl-d-glucosamine-specific, l-fucose-specific, and α-2,3 sialic acid-specific legume lectins with very similar binding profiles^28,29^ Cocrystallization also revealed that Jacalin binding involves a network of specific interactions within its three subsites. The primary site relies on a combination of aromatic, acidic, and polar amino acids, along with the protein backbone and the free amino terminus. These residues form hydrogen bonds with the sugar, typically galactose or *N*-acetyl-d-galactosamine.^30^

A recurring theme in lectin crystallography is its potential to guide future studies. For example, structural studies of viral lectins, including the hemagglutinin protein of influenza, have informed the design of glycan-based inhibitors and vaccines.^31–33^ Similarly, the crystallographic analysis of tumor-associated lectins has highlighted their roles in cancer progression and immune evasion, providing new possibilities for therapeutic intervention^34,35^ As we consider these applications, it becomes clear that lectin crystallography not only advances basic science but also allows for translational research.

Despite these advances, challenges remain. One notable limitation is the difficulty in crystallizing certain lectins, particularly those that are membrane-bound or exhibit significant conformational flexibility or extensive glycosylation. In our assessment, the integration of computational modeling with experimental approaches holds immense promise in overcoming these barriers. Machine learning algorithms can now predict glycan binding affinities and generate models of protein-glycan interactions, accelerating hypothesis-driven research. This computational synergy is poised to reshape the field, enabling us to explore the vast diversity of lectins and their interactions with unparalleled precision.

Thus, X-ray crystallography has been essential in defining lectin structures and their interactions, allowing for deeper functional understanding and more potential applications, while also highlighting areas where new integrated approaches are necessary.

### 1.3 Proteus 2.0

Proteus, the ever-shifting sea deity, son of Poseidon, possessed the power to assume myriad forms, from beast to element. His metamorphic abilities allowed him to elude capture, transforming into animal, water, or fire at will. Yet, when held fast, he revealed truths of past, present, and future. The adjective “protean”, signifying changeability, stems from his fluid nature. Proteins, too, are protean, characterized by an inherent flexibility and capacity for multiple conformations. This inherent changeability in protein structure, and how it relates to function, has been a central theme of our research for decades.

Our initial exploration of this theme, published in 2001,^36^ focused on Diocleinae lectins, a family of proteins closely related to ConA, and examined their diverse biological activities despite their high structural similarity. In our paper, “Revisiting Proteus: Do Minor Changes in Lectin Structure Matter in Biological Activity? Lessons from and Potential Biotechnological Uses of the Diocleinae Subtribe Lectins”, we highlighted how subtle variations in carbohydrate-binding sites and quaternary structures significantly influenced these lectins’ interactions with complex carbohydrates and cell surface receptors, ultimately shaping their immunomodulatory functions. A key finding was that minor amino acid substitutions, even those distant from the active site, can dramatically alter carbohydrate recognition and downstream biological effects, leading to divergent capacities to stimulate cytokine production, induce histamine release, and modulate macrophage activity. The quaternary structure of these lectins, particularly their pH-dependent dimer-tetramer equilibrium, further exemplifies their structural plasticity, affecting multivalency and the ability to cross-link cellular receptors, thereby modulating signal transduction and immune responses. This pH-dependent oligomerization, a critical feature for many ConA-like lectins, arises from the protonation state of key histidine residues at subunit interfaces, altering interdimer contacts and shifting the equilibrium between dimeric and tetrameric forms under different pH conditions.^37^ Their ability to interconvert between structural forms under varying conditions is a testament to their functional adaptability, reinforcing the Proteus metaphor and extending it to biotechnological applications, where these lectins are explored as immunomodulators, insecticidal agents, vehicles for targeted drug delivery among many other applications.

Now, two decades later, we revisit Proteus. Our laboratory’s ongoing work in lectin crystallography has, once again, embodied this metaphor of transformation and adaptability. Like the shape-shifting god, our explorations of lectin structures have evolved alongside advancements in the field, continually revealing new facets of legume lectin biology. This enduring metaphor encapsulates our decades-long commitment to unraveling the complexities of lectin biology, particularly through the lens of structural biology.

Within this context, this review charts two decades of lectin crystallography at the BioMol Lab, from initial challenges to breakthroughs in our understanding of legume lectin structures and activities. It highlights key advancements, the unique contributions stemming from the study of Brazilian biodiversity, lingering questions, and emerging trends, ultimately setting the stage for the future of lectin research. We will how the “Protean” nature of lectins, their structural flexibility and functional diversity, has been a recurring theme in BioMol-Lab’s research, mirroring the god’s own shape-shifting abilities.

The Proteus metaphor serves as a guiding principle for this review, emphasizing the structural adaptability of lectins and how understanding these subtle changes is key to deciphering their diverse functions, a central focus of BioMol-Lab’s research narrative.

## 2 Lectin Structures within BioMol-Lab: a 20-Year Odyssey

The primary objective of this section is to chronicle the evolution of lectin crystallography at BioMol-Lab, from its foundational phase reliant on external support to its current status as an independent research center, highlighting key structural discoveries and methodological advancements along the way.

### 2.1 Humble Beginnings

The early days of BioMol-Lab in the field of lectin crystallography were marked by significant challenges. The landscape of protein crystallography in Brazil at the time was, it could be said, nascent. In fact, an “underdeveloped” state would be a more accurate description. Driven by ambitious scientific goals and a degree of calculated risk-taking, we dreamed big, but resources were scarce and expertise in protein crystallography was virtually nonexistent in the Northeast region of Brazil. Our infrastructure was modest, and the idea of determining three-dimensional structures of proteins seemed, to many, a distant fantasy.

It is crucial to acknowledge, however, that although dedicated resources for macromolecular crystallography were particularly scarce in the Northeast at the time, the broader field of X-ray crystallography in Brazil was already established and undergoing significant development. A central figure in this process was Prof. Yvonne Primerano Mascarenhas, who trained under George A. Jeffrey and Brian Craven at the University of Pittsburgh (1959–1960) before returning to Brazil to found the country’s first X-ray crystallography laboratory at USP São Carlos in the early 1960s. She also played a key role in the creation of the Brazilian Crystallographic Association (ABCr) in 1971. Her mentorship led to the formation of a cohort of prominent crystallographers, including Prof. Aldo Craievich, whose training at LURE (France) allowed him to conceptualize and lead the development of the Laboratório Nacional de Luz Síncrotron (LNLS), the first synchrotron facility in the Southern Hemisphere. Prof. Glaucius Oliva contributed significantly to the expansion of structural biology in Brazil, particularly through its application to drug discovery for neglected diseases at institutions such as CIBFar (USP). Additionally, Prof. Richard Charles Garratt was instrumental in consolidating protein crystallography infrastructure at USP São Carlos from the early 1990s onward, with a focus on macromolecular structure determination. These coordinated efforts were critical to the establishment of Brazil’s national capacity in structural biology and to the eventual development of advanced synchrotron resources, which are now essential for the work conducted at BioMol-Lab. Reflecting this scientific lineage, Dr. Walter Filgueiras, whose contributions to our initial crystallographic studies are acknowledged later in this review, completed his PhD under Prof. Mascarenhas.

Dependence on international collaborations was, therefore, a necessity. The group of Prof. Dr. Juan J. Calvete, at the Instituto de Biomedicina de Valencia, Consejo Superior de Investigaciones Científicas (CSIC), and the laboratory of Prof. Dr. Antonio Romero, at the CSIC in Madrid, Spain, played fundamental roles in this initial stage. These collaborations were invaluable for the training of human resources and for the execution of the first lectin crystallization experiments. Through these interactions, there was an opportunity to gain hands-on experience in crystallography and X-ray diffraction techniques.

The research within our lab has long been dedicated to exploring the rich biodiversity of Brazilian plants, with a particular focus on lectins. One of our earliest successes, which became the logo of BioMol lab, involved the crystal structure of the C. brasiliensis lectin (ConBr) in 1997 (PDB ID 1AZD—Figure 1A). ConBr is a close relative of the well-researched Concanavalin A (ConA), and although they share high sequence similarity, ConBr exhibited distinct biological activities. Specifically, we knew that ConBr demonstrated a greater potency than ConA in stimulating lymphocyte proliferation and inducing the production of cytokines, such as TNF-α and IFN-γ, by human peripheral blood mononuclear cells. Using ConA as a model for molecular replacement, we solved the ConBr structure and identified key amino acid differences that might explain these functional differences. One significant variation is the substitution of Gly58 in ConBr with Asp58 in ConA. Although this residue is located away from the carbohydrate-binding site, it plays a crucial role in the quaternary structure of the lectin. In ConA, Asp58 forms a hydrogen bond with Ser62 of another subunit, contributing to a more compact tetrameric structure. However, in ConBr, the presence of Gly58 prevents the formation of this hydrogen bond, leading to a more “open” quaternary structure. This altered conformation affects the relative orientation of the carbohydrate-binding sites, potentially influencing the lectin’s ability to interact with and cross-link cell surface receptors. These subtle structural and binding differences likely contribute to the observed variations in biological activity. This work marked our first major contribution to understanding structure–function relationships in lectins, a puzzle that, surprisingly, remains an active area of research to this day. The publication detailing the ConBr structure was selected as the cover of FEBS Letters and other research articles from our laboratory have also been similarly highlighted on journal covers.^38–40^

**Figure 1:**
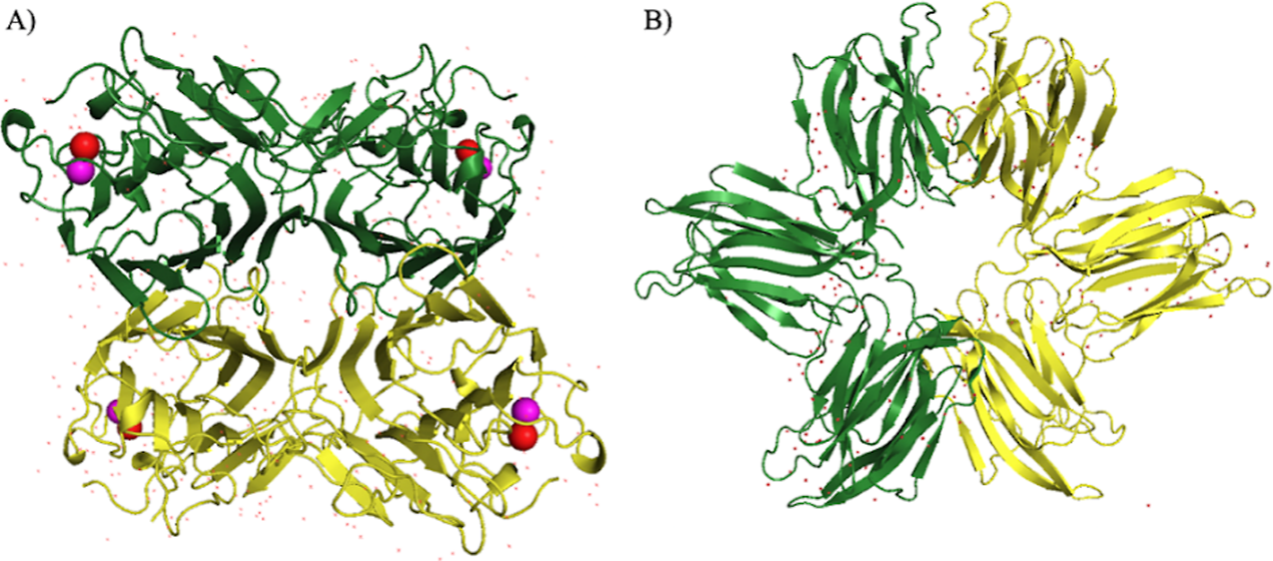
Relevant 3D structures solved in BioMol-Lab. (A) Crystal structure of Canavalia brasiliensis lectin (ConBr, PDB ID: 1AZD), the first protein structure solved by BioMol-Lab, and (B) structure of Parkia platycephala lectin (PPL, PDB ID: 1ZGS), the first structurally characterized lectin from the Mimosoideae subfamily. Structures are shown as cartoon representations with monomers in green and yellow; calcium and manganese ions are represented as red and pink spheres, respectively.

Driven by these initial findings and fueled by collaborations with Spanish groups, we expanded our studies to other Diocleinae lectins. This included work on Dioclea guianensis lectin, where we solved structures of both the native and a Cd/Cd-substituted form, revealing details about the metal-binding site and the structural diversity within the Diocleinae lectins (PDB id: 1HP9) With Cratylia floribunda lectin (CFL), we focused on understanding the pH-dependent dimer-tetramer transitions. We solved structures of different oligomeric states at different pH values (PDB ids: 2D3R and 2D3P), providing valuable insights into the mechanism behind these transitions. This work revealed that CFL exists as a tetramer at pH 7.5 but transitions to a dimer at pH 4.6. This transition is physiologically significant as it affects the lectin’s valency and, consequently, its ability to cross-link cell surface receptors and trigger downstream signaling events. We discovered that a single amino acid difference when comparing CFL to D. guianensis lectin (DGL), specifically the substitution of Asn (in CFL) for His (in DGL) at position 131, drastically alters interdimer contacts. In DGL, His131 forms strong hydrogen bonds across the dimer interface, stabilizing the tetrameric form even at low pH. However, in CFL, the Asn at position 131 disrupts these interdimer contacts, leading to a pH-dependent dimer-tetramer equilibrium.

We also ventured into the field of recombinant lectin expression. We successfully expressed functional recombinant lectins from D. guianensis and Dioclea grandiflora in E. coli, a significant achievement due to their complex post-translational processing. These lectins undergo the rare circular permutation processing that includes propeptide cleavage and relinking, which are difficult to replicate in the bacterial expression system. Overcoming these challenges opened up new possibilities for research, enabling us to study a wider range of lectins and engineer mutants to probe structure–function relationships.^41^ For instance, we converted the pH-stable tetrameric D. grandiflora lectin into a structure exhibiting pH-dependent dimer–tetramer transition, demonstrating the power of combining recombinant protein technology with X-ray crystallography (PDB ids: 2JE7, 2JDZ, 2JEC, and 2JE9).

Our exploration of Brazilian biodiversity also led us to the Parkia genus, where we characterized two intriguing proteins: PPL2 and PPL. PPL2, a Chitinase with lectin-like properties, is one of the few Chitinase-related agglutinins with a solved 3D structure (PDB id: 2GSJ). The PPL2 structure revealed a compact (β/α)­8 barrel, a fold typically found in enzymes, and we demonstrated that it did indeed possess Chitinase activity, albeit very weak. PPL, the first structurally characterized Mimosoideae lectin (PDB id: 1ZGS—Figure 1B), was a noteworthy achievement. Its structure revealed a novel circular arrangement of β-prism domains, with a monomer comprising three Jacalin domains in tandem. This unique arrangement suggests a new mechanism for multivalent lectin interactions, distinct from other Jacalin-related lectins, which typically exhibit a structure with one β-prism domain per monomer. This work marks the first reported structural analysis of lectins from plants belonging to the Mimosoideae subfamily of legumes a part of the interesting group of tandem repeat lectins.^42^

However, this phase was also characterized by limitations inherent to the dynamics of international collaborations. The collaborators, with their own research agendas, naturally prioritized the crystallization of proteins relevant to their projects. Consequently, the pace and direction of research in lectin crystallography at BioMol-Lab were, to some extent, influenced by external factors. This dynamic, while understandable, presented challenges to autonomously directing the research focus at BioMol-Lab. Therefore, we needed to take a leap forward, to step out of the shadows and build our own expertise in lectin crystallography here, in Brazil, focusing on our rich and unexplored natural sources.

Despite these limitations, this initial phase was crucial for the subsequent development of lectin crystallography at BioMol-Lab. The experience gained, the knowledge absorbed, and the techniques learned during this period served as the foundation upon which the laboratory built its independent research capacity. The collaborations with the Spanish groups, although focused on specific objectives, allowed the formation of an initial critical mass and the establishment of a research culture in protein crystallography at BioMol-Lab. In summary, this foundational period yielded the first lectin structures from BioMol-Lab, including ConBr and novel Mimosoideae subfamily lectins. These early successes provided critical insights into structure–function relationships, such as the molecular basis for differing biological activities in highly similar lectins and the mechanisms of pH-dependent oligomerization, thereby laying the crucial groundwork for the laboratory’s future autonomy and research directions.

### 2.2 Habemus Crystallum: The Journey toward the “Salinha”

The journey toward establishing a robust lectin crystallography program at BioMol-Lab was not solely a solitary endeavor. Serendipity, combined with strategic collaborations, played a crucial role in propelling us forward. This period could be considered our second phase, where we transitioned from a state of complete dependence to one of burgeoning autonomy, aided, in no small part, by some unexpected assistance.

A pivotal moment arrived with an unforeseen opportunity. Surplus funds from a CNPq bioinformatics project became available, an event that allowed us to acquire our first Silicon Graphics workstation. This acquisition was a significant upgrade to our computational capabilities and would prove instrumental in our future work. Recognizing the potential of these funds to further our crystallographic ambitions, we requested and were granted permission to relocate them. This enabled the construction of a modest, semisubterranean laboratory space, which came to be known as the “salinha” (little room), depicted in Figure 2. It was here, through the diligent efforts of Plinio Delatorre, then pursuing his doctorate in our lab, that our crystallography efforts truly began to take root. The “salinha” became a symbol of our growing independence and a hub for our crystallographic work. The lab’s inauguration was even featured in national news outlets such as Folha de São Paulo and Agência FAPESP, highlighting its status as the first protein crystallization laboratory outside the state of São Paulo, a point that pinpointed the lab’s pioneering role in decentralizing advanced scientific research in Brazil.

**Figure 2:**
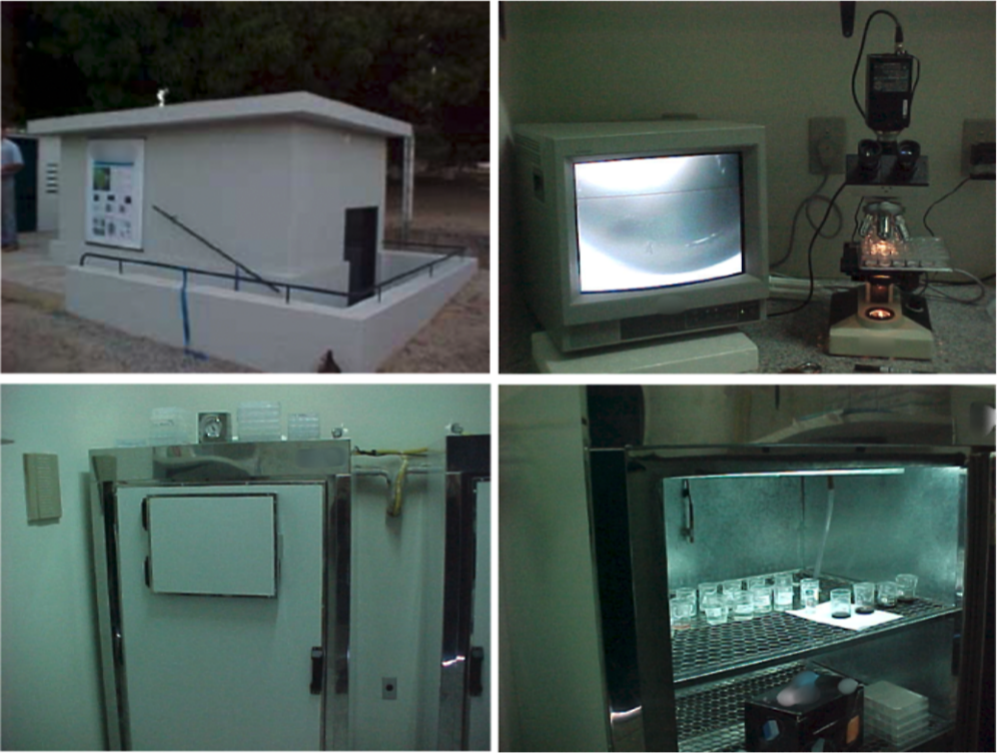
Images of the “salinha” and its equipment during its establishment. Images courtesy of the authors.

The establishment of our protein crystallization facility attracted significant attention. As reported in an August 12, 2003 article by Agência FAPESP, the new laboratory, fully funded by CNPq, was recognized as a vital addition to the national scientific infrastructure, “accrediting the State of Ceará as one of the central headquarters of the National Proteomic Network”, as stated by one of us (B.S.C.) during the interview. The Folha de São Paulo article, published on August 15, 2003, further emphasized the unique nature of our lab, noting our focus on plant proteins and pioneering work in isolating lectin structures from marine organisms, including algae.

In those early days, expertise in protein crystallography was still scarce in Brazil, and our group faced significant limitations in personnel and infrastructure. At this critical moment, we were fortunate to receive essential support from Dr. Walter Filgueiras, a crystallographer at UNESP, who generously shared his knowledge and experience to help us navigate the complexities of the technique. His contribution was instrumental in setting up our initial crystallization trials and troubleshooting the challenges that inevitably arose. Through his guidance, we were able to demonstrate that, despite the overall complexity of structure determination (which involves not only crystallization but also extensive data collection and analysis), valuable work could be accomplished with modest resources. Dr. Filgueiras became a key collaborator during this formative period.

This period was also marked by our lab’s commitment to building local expertise. The “salinha”, equipped with our Silicon Graphics workstation and fueled by the repurposed bioinformatics funds, became a training ground for the first generation of protein crystallographers at BioMol-Lab. With only 50,000.00 Brazilian Reais from CNPq, we were able to start the work of protein and amino acid crystallography. Before this, all the material researched at the university was sent to institutes in Spain. Our lab comprised seven doctors and 30 scholarship holders (master’s, doctorate and bachelor training). These individuals, trained within our fledgling program, would go on to play a vital role in the future of lectin crystallography, not only within our lab but also in the broader Brazilian scientific landscape. This period of collaborative growth was very important for our future success, demonstrating that with a combination of strategic resourcefulness, external support, and a commitment to training, even the most ambitious scientific goals can be achieved. The investment was a bargain, especially when compared to the alternative: “paying dearly in royalties to use what is ours”, a perspective previously articulated by one of us (B.S.C.) and still considered valid.

#### 2.2.1 Early Scientific Breakthroughs from an Independent BioMol-Lab

Since this period saw a shift in the lectin field toward more structurally focused research, each solved structure became highly valuable, and we took full advantage of this trend. One of the key lessons reaffirmed during this time was that small structural variations can lead to profound functional consequences. This was exemplified by the discovery of a novel binding site, a hidden compartment that accommodates α-aminobutyric acid (Abu), a nonprotein amino acid. This binding pocket was not an isolated feature of Canavalia gladiata lectin (CGL) (PDB id: 1WUV), but appears conserved in several legume lectins, including Canavalia maritima lectin (ConM) and Cymbosema roseum lectin I (CRLI), suggesting a broader, previously unrecognized role. It has been proposed that these lectins may be involved in transporting or regulating secondary metabolites potentially contributing to plant defense mechanisms.^43,44^ This aligns with the idea that lectins could act as triggers for induced systemic resistance, opening new research avenues. Beyond Abu, other “non-canonical” binding sites in legume lectins have been identified, broadening our understanding of their functional roles beyond carbohydrate recognition. For instance, indole-3-acetic acid (IAA), an essential plant hormone (auxin) involved in growth and development, binds within a hydrophobic cavity of several ConA-like lectins, including C. maritima lectin, suggesting a role in auxin regulation and plant growth control.^45^ Collectively, these findings reveal that legume lectins possess a wider range of binding capabilities than previously thought, interacting with diverse molecules and potentially modulating various physiological processes in plants.

It gets more complex when we consider the remarkable ability of many Diocleinae lectins to modulate nitric oxide (NO) production, a key signaling molecule in various physiological processes, including vasodilation and inflammation. The work on ConM, Dioclea virgata lectin (DvirL), and ConBr (PDB ids: 2CWM, 3RRD and 4H55) collectively demonstrates a compelling correlation between the structural features of the CRD and the capacity to induce NO production in biological models. We could verify that specificity is one of the factors; just as important is the way they bind. A smaller CRD volume, a specific spatial arrangement of key amino acid residues, these small variations appear to fine-tune the interaction with cell surface glycans, influencing the biological effect. Further, investigations into ConM’s interactions with molecules like GABA and adenine, linked to its insecticidal properties, provide a structural basis for its role in plant defense (PDB id: 4I30). The intricate relationship between ConM and these molecules, revealed through X-ray crystallography, suggests a mechanism where ConM might act as a delivery system of molecules to insects. This detailed understanding, coupled with findings on the lectin carbohydrate binding specificity, as seen in PDB id: 4I30, uncovers the potential of lectins in biotechnological applications, particularly in developing novel pest control strategies. The route-dependent inflammatory activity of CRLI adds another dimension to this complex interplay. It highlights that the context of lectin-cell interactions and the specific cellular environment can dictate the nature of the response, revealing the versatility of these proteins, but further complicating the definition of structure/function relationships. This duality in CRLI’s activity, being both pro- and anti-inflammatory, is a notable phenomenon, although it has been reported for the first time much earlier, in 1997.^46^

The pH-dependent oligomerization behavior of lectins has been revisited during the structure solving of Canavalia boliviana lectin (Cbol) (PDB id: 4K20, 4K21, 4K1Y, 4K1Z), and the finding that its anti-inflammatory properties stem from its carbohydrate-binding domain further strengthens the structure/function connection.

This phase, centered around the “salinha” and local collaborations, thus marked BioMol-Lab’s significant move toward research autonomy. Key scientific outcomes included the characterization of noncanonical binding sites for some legume lectins, and an enhanced understanding of how subtle structural variations in highly similar proteins correlate with complex biological responses, including nitric oxide modulation and route-dependent inflammatory effects, further illustrating the “Protean” functional adaptability of these proteins.

### 2.3 Standing on Our Own Two Feet: New Beginnings

The “salinha”, once a symbol of our nascent independence, had served its purpose admirably. As our collaborators, like Dr. Walter Filgueiras and the teams in Spain, naturally progressed with their own research endeavors, we were faced with a challenge. The momentum we had built in lectin crystallography needed to be sustained, and even accelerated, here in Brazil. The previously described underdeveloped conditions of Brazilian crystallography were slowly changing, but the structural biology area within the lab needed to maintain its progress. It was time to transition from a phase of nurturing growth to one of true autonomy and leadership. While the departure of close collaborators marked a shift, it did not signal an end to collaboration altogether. Strategic partnerships remained vital, and a particularly fruitful one blossomed with Prof. Dr. Karl Gruber’s group at the University of Graz, Austria. This collaboration provided crucial access to expertise in advanced crystallographic techniques and data analysis, particularly in the structure of Vatairea macrocarpa lectin, a very important galactose-specific Dalbergiae lectin in the context of our lab. Although the collaboration focused on a single plant lectin, it was a particularly important one.

This new phase demanded a significant investment in technology to solidify our independence. Recognizing that high-throughput crystallization screening was essential for maximizing our chances of success, we acquired a Mosquito liquid handling robot from TTP Labtech. This was a substantial acquisition, but represented a major leap forward in our capabilities, enabling the setup of thousands of crystallization trials with minimal protein and crystallization condition consumption, a critical advantage when working with precious samples extracted from rare Brazilian flora. The Mosquito became a cornerstone of our workflow, dramatically increasing our efficiency and allowing us to explore a wider range of crystallization conditions. Perhaps most importantly, the support of the Laboratório Nacional de Luz Síncrotron (LNLS), and subsequently, the state-of-the-art Sirius synchrotron, provided access to high-intensity X-ray beams and their expertise in crystal diffraction and structure solving. This access was, and continues to be, absolutely essential for collecting high-quality diffraction data.

As previously mentioned, the collaboration with Prof. Dr. Karl Gruber’s lab at the University of Graz was very important. This partnership allowed the definition of the structure from the glycoprotein V. macrocarpa lectin (VML), a protein with significant potential in cancer research due to its affinity for the Tn antigen, a tumor-associated carbohydrate. Our collaborative efforts yielded high-resolution crystal structures of VML bound to both the Tn antigen and its monosaccharide component, GalNAc (PDB id: 4U2A and 4U36). These structures provided the first detailed view of a Dalbergieae lectin interacting with this tumor-associated antigen. Building on this, the structure of a recombinant form of VML (rVML) (PDB id: 4XXA, 4WV8 and 4XTM) was determined. This addressed the issue of heterogeneity often found in plant-derived lectins. We determined structures of rVML with GalNAc, Tn antigen, and lactose, confirming that the recombinant protein retained the binding specificity and tetrameric structure essential for its biological activity. Using these structures also expanded our computational analysis and we found that rVML, like other Tn-binding lectins, preferred mucins with a higher density of Tn antigen, but its unique arrangement of charged and hydrophobic residues near the CRD conferred distinct binding characteristics. It is important to mention that, including the structure of VML, BioMol-Lab has made pioneering contributions to the structural study of legume lectins from the Dalbergiae tribe.

In this phase, which is actively ongoing, our ambitions extended beyond simply solving additional structures. We recognized that the field was maturing and made use of the powerful integration of X-ray crystallography with computational techniques to maintain research excellence. Our goal was to move beyond static structural descriptions and to understand lectin structures in a dynamic, evolutionary, and functional context.

This integration manifested primarily through the following approaches:

•Sequence-Structure Correlation and Predictions: We actively used sequence alignments, guided by our growing database of lectin structures, to predict structural features before crystallization. If a particular residue was invariably conserved across lectins with a known structure, we could confidently predict its structural role. Conversely, variations in sequence, particularly within or near the CRD, could be used to hypothesize about differences in carbohydrate specificity. This approach was crucial for understanding structure–function relationships as seen in.^47,48^

•Homology Modeling for Structure Prediction: When crystallization proved difficult, we employed homology modeling, using our existing Diocleinae structures as templates. This was particularly valuable for lectins from less-readily available plant sources. The Dioclea lasiophylla lectin (DlyL) study exemplifies this. An initial homology model^49^ provided crucial insights into the likely overall fold and the location of key functional regions, guiding our subsequent, successful crystallization efforts (PDB ID: 6CJ9). The eventual crystal structure validated the model, demonstrating the power of this approach.
•Molecular Docking: We applied molecular docking with various programs to explore lectin interactions in silico. While cocrystallization provided snapshots of binding with specific ligands, docking allowed us to test a much wider range of carbohydrates and small molecules. This was particularly useful for investigating the potential binding of more complex glycans that were difficult to cocrystallize. For example, in the later study of Canavalia villosa lectin (PDB ID: 8SZO), the crystal structure, combined with docking simulations, provided a detailed understanding of its interactions with various mannose-containing glycans.^50^
•Molecular Dynamics Simulations: Within BioMol-Lab, we employ molecular dynamics (MD) simulations as a key computational tool to complement the static nature of X-ray crystal structures. Recognizing that crystal structures offer a snapshot, MD allows us to explore the dynamic behavior of our lectins over time. These simulations provide further insights into protein flexibility (particularly in loops), conformational changes, and ligand interactions, which are crucial for generating new hypotheses and guiding future experimental research. For example, MD simulations applied to investigate the effects of circular permutation by comparing unprocessed ProConA with mature ConA provided crucial insights. These simulations demonstrated that ProConA processing significantly enhances the structural stability of the mature lectin, particularly in maintaining the tetrameric oligomer, while having minimal impact on its intrinsic carbohydrate-binding properties.^51^ While powerful, these simulations are always interpreted in light of their inherent methodological limitations, such as force field accuracy and the challenge of achieving exhaustive conformational sampling. The focus in this context is on how such MD-derived insights complement structural data to generate new hypotheses and effectively guide future experimental research directions.
•Database Integration: We actively utilized protein structure databases (primarily the PDB) as active research tools. We routinely compared our new structures to existing ones, searching for structural motifs, identifying subtle differences that might have functional significance, and placing our work within the broader context of lectin structural biology.

This current phase, characterized by technological self-sufficiency and the synergistic use of crystallography and computational methods, has enabled BioMol-Lab to tackle more complex structural questions, delve deeper into structure–function relationships, and make significant contributions such as the structural elucidation of Dalbergiae lectins and their interactions with tumor antigens. The integration of techniques like MD simulations, homology modeling, and docking has become central to dissecting the “Protean” nature of lectins. Table 1 summarizes the structures of the proteins obtained by our group and collaborations.

**Table 1:** Summary of Structures of Lectins Solved in the BioMol Lab^a^

lectin	PDB IDs	year range	fold class/quaternary assembly	key findings/biological relevance
Canavalia brasiliensis seed lectin (ConBr)	1AZD, 3JU9, 4H55, 4P14, 4PCR	1997–2015	legume lectin (β-sandwich); tetramer	quaternary structure differences explain distinct bioactivity vs ConA; structural basis for eNOS activation and NO release; noncanonical ligand (alpha-aminobutyric acid, adenine, gamma-aminobutyric acid, beta-d-ribofuranose) binding sites suggest broader physiological roles
Dioclea guianensis seed lectin (DGuiL)	1H9P, 1H9W, 2JDZ, 2JE7	2001–2008	legume lectin (β-sandwich); tetramer	novel Mn^2+^ binding site (S5) identified; structural basis for pH-dependent dimer-tetramer equilibrium elucidated (linked to Asn131 and interdimer contacts); active recombinant form produced, aiding in mechanistic studies of oligomerization
Canavalia gladiata lectin (CGL)	1WUV, 2D7F, 2EF6, 2OVU, 2P2K	2006–2007	legume lectin (β-sandwich); tetramer	identified a novel, conserved binding site for alpha-aminobutyric acid, suggesting a role in secondary metabolite transport/plant defense; detailed carbohydrate (α-methyl-mannoside, various dimannosides) binding modes revealed differences from ConA/ConM, offering insights into structure–activity relationships in Diocleinae lectins
Parkia platycephala seed lectin (PPL)	1ZGR, 1ZGS	2005–2006	Jacalin-related (β-prism trimer per monomer); dimer of trimers	first Mimosoideae lectin structure; revealed novel toroid-shaped dimer with three β-prism domains per monomer, each showing different mannose binding modes; suggests mechanism for multivalent interactions in plant defense
Canavalia maritima lectin (ConM)	2CWM, 2CY6, 2CYF, 2OW4, 2P34, 2P37, 3SNM, 4DPN, 4I30, 4TYS, 4TZD, 5BYN	2005–2015	legume lectin (β-sandwich); tetramer	revealed structural basis for NO-releasing vasorelaxant activity; detailed binding modes for disaccharides (trehalose, maltose) and various dimannosides, explaining differential affinities and structure–activity relationships; identified noncanonical binding sites for indole-3-acetic acid (suggesting role in hormone regulation) and other diverse small molecules (e.g., GABA, adenine, resveratrol)
Parkia platycephalla lectin 2	2GSJ	2006	chitinase ((β/α)8-barrel); Monomer	high-resolution structure of a bifunctional protein with *N*-acetylglucosamine-specific lectin and endochitinase (GH18 family) activities; (β/α)8 barrel fold with identified catalytic residues; suggests a dual role in plant defense
Cratylia floribunda seed lectin (CFL)	2D3P, 2D3R	2007	legume lectin (β-sandwich); tetramer	elucidated structural basis of pH-dependent dimer-tetramer transition by comparing acidic (dimer) and basic (tetramer) pH structures; identified key residues (e.g., His51, Asn118, Thr120, Asn131) and loop conformational changes (117–120) critical for modulating quaternary assembly and explaining differences from pH-independent tetramers like DGL
Dioclea violacea seed lectin (DVL)	2GDF, 3AX4	2006–2013	legume lectin (β-sandwich); tetramer	crystal structure (complexed with 5-bromo-4-chloro-3-indolyl-α-D-mannopyranoside) revealed a narrow, deep CRD; this geometry was correlated with its moderate vasorelaxant activity (43%) compared to DRL, linking subtle CRD differences to biological potency
Lotus tetragonolobus seed lectin (LTL)	2EIG	2008	legume lectin (β-sandwich); tetramer	revealed a novel legume lectin tetrameric assembly (a new arrangement of two back-to-back GS4-like dimers), influenced by *N*-glycosylation at Asn4 and distinct dimer–dimer interactions; provided insights into its fucose-specific binding site, crucial for understanding its interaction with l-fucosyl carbohydrates
Dioclea rostrata lectin (DRL)	2ZBJ	2008	legume lectin (β-sandwich); tetramer	crystal structure; provided structural basis for understanding its distinct pH-dependent oligomerization and carbohydrate recognition. Comparative studies linked its wider, shallower CRD to potent vasorelaxant activity
Bothrops neuwiedi venom phospholipase	3MLM	2011	sPLA2 group II fold; Monomer	structure of Lys49-PLA2 myotoxin (Bn IV) with myristic acid in its catalytic site; identified a C-terminal heparin-binding domain (NKKYRY) critical for myotoxicity, suggesting a heparin-mediated cell signaling mechanism for membrane damage, independent of catalytic activity or dimer formation
Cymbosema roseum seed lectin (CRL)	3A0K, 4MYE	2011–2014	legume lectin (β-sandwich); tetramer	structure (1.8 Å) elucidated to understand its dual, route-dependent pro- (local) and anti-inflammatory (systemic) activities; confirmed tetrameric assembly and an alpha-aminobutyric acid binding site. Differences in carbohydrate binding domain design (e.g., Tyr12, Glu205) proposed as basis for its distinct biological responses
Camptosema pedicellatum seed lectin (CPL)	3U4X	2012	legume lectin (β-sandwich); tetramer	structure with X-Mannose revealed a conservative mutation in CRD’s hydrophobic subsite, demonstrating how subtle changes in this region impact H-bonding and modulate carbohydrate recognition
Platypodium elegans lectin (PELa)	3ZVX, 5U38	2012–2018	legume lectin (β-sandwich); dimer	unusual specificity for asymmetrical complex *N*-glycans (structural basis determined); native form is the first Dalbergieae lectin shown to exhibit CRD-dependent hypernociceptive activity
Parkia biglobosa seed lectin (PBL)	4MQ0	2014	Jacalin-related (β-prism trimer per monomer); dimer of trimers	structure revealed a Jacalin-related fold with three tandem β-prism domains per monomer, similar to PPL; comparative studies and docking highlighted differences in carbohydrate interaction energies, contributing to structure–activity understanding in Parkia lectins
Dioclea sclerocarpa lectin (DSL)	4NOT	2015	legume lectin (β-sandwich); tetramer	structural analysis explained its low vasorelaxant activity (cf. other Dioclea lectins) due to a less favorable CRD design, particularly identifying Glu205 as a key residue diminishing this effect
Canavalia grandiflora seed lectin (CgranL)	4L8Q	2014	legume lectin (β-sandwich); tetramer	structure (with X-Man modeled) correlated with its moderate NO-mediated vasorelaxant activity; analysis of CRD residue distances provided insights into structural factors differentiating vasorelaxant efficacy among Canavalia lectins
Vatairea macrocarpa seed lectin (VML)	4U2A, 4U36, 4WV8, 4XTM, 4XTP, 4XXA	2014–2016	legume lectin (β-sandwich); tetramer	high-resolution structures of native and recombinant VML with Tn antigen (GalNAc-α-*O*-Ser) and GalNAc elucidated detailed binding modes; revealed that subtle CRD features (hydrophobic/charged clusters) govern recognition of complex tumor-associated antigens, establishing VML as a promising tool for cancer research
Centrolobium tomentosum seed lectin (CTL)	5EYX, 5EYY	2016	legume lectin (β-sandwich); dimer	first structure of a Centrolobium genus lectin (mannose/glucose-specific dimer) with pro-inflammatory activity; elucidated CRD, metal/glycosylation sites, and basis for mannose/glucose binding
Canavalia virosa lectin (ConV)	5F5Q	2016	legume lectin (β-sandwich); tetramer	structure revealed typical Diocleinae fold; demonstrated CRD-dependent pro-inflammatory activity and cytotoxic effects against C6 glioma cells (mitochondrial disruption), suggesting anticancer potential
Pisum arvense lectin (PAL)	5T7P	2017	legume lectin (β-sandwich); dimer	structure of a Vicieae tribe lectin; detailed CRD analysis (docking/MD) revealed differential affinities for carbohydrates based on linkage/orientation and characterized *N*-glycan interactions, a first for this tribe
Dioclea reflexa seed lectin (DrfL)	5TG3	2017	legume lectin (β-sandwich); tetramer	high-resolution structure (1.765 Å); exhibited low-intensity NO-mediated vasorelaxant activity involving its CRD; in silico studies showed strong *N*-glycan interaction, highlighting unique CRD properties among diocleinae lectins
Canavalia bonariensis seed lectin (CaBo)	5U3E	2018	legume lectin (β-sandwich); tetramer	structure with X-Mannose detailed typical legume lectin features; demonstrated significant in vitro antiglioma activity (decreased viability/migration via autophagy and cell death) mediated by *N*-glycan interaction
Dioclea lasiocarpa seed lectin (DLL)	5UUY	2017	legume lectin (β-sandwich); tetramer	structural characterization revealed typical legume lectin features; demonstrated potent antiglioma activity (C6 cells) through caspase 3-mediated apoptosis, highlighting its potential in cancer research
Dioclea lasiophylla seed lectin (DlyL)	6CJ9	2018	legume lectin (β-sandwich); tetramer	structure with X-Man characterized; showed antiglioma potential (C6 cells: impaired migration, autophagy, caspase 3-mediated cell death); unique preference for complex/hybrid *N*-glycans cf. other Diocleinae lectins
Canavalia villosa lectin (Cvill)	8SZO	2023	legume lectin (β-sandwich); tetramer	structural characterization and fine carbohydrate specificity (mannose-specific) elucidated; demonstrated antiproliferative activity against cancer cells, mediated by carbohydrate binding, cellular uptake, and concomitant activation of autophagy and apoptosis
Dioclea megacarpa lectin (DmegA)	8UX7	2023	legume lectin (β-sandwich); tetramer	crystal structure determined in complex with X-Man (2.20 Å), providing structural data for this Diocleinae lectin
Centrolobium microchaete seed lectin (CML)	9C4I	2024	legume lectin (β-sandwich); dimer	high-resolution structure (1.3 Å) with a methylated dimannoside detailed a dimeric Dalbergieae lectin; showed limited in vitro antiglioma activity (high concentration, autophagy activation), potentially due to its dimeric nature and unusual asymmetric glycan specificity

a The table lists lectins, their associated ligands, the time range of structural determination, and corresponding PDB IDs.

## 3 Key Structure–Function Insights from Two Decades of Legume Lectin Research at BioMol-Lab

Having recounted BioMol-Lab’s journey through two decades of lectin crystallography in Section 2, this section now distills the cumulative understanding of key structure–function relationships in legume lectins that emerged from our work, contextualized by established knowledge in the field.

Our experiences have consistently shown how conserved structural motifs and subtle variations dictate their diverse biological roles. Working for so long on the structure of lectins, we learned that the surprises are often found in the details. Our consistent observation across the numerous legume lectin solved at BioMol-Lab (many of which are detailed in Table 1 and discussed in Section 2) confirmed the highly conserved architecture typical of this family. Legume lectins generally have amino acid sequences of 230–260 residues (25–30 kDa per monomer), assembling into a tertiary structure of antiparallel beta-strands that form two beta sheets. These sheets are organized one over the other, creating the characteristic beta-sandwich fold (illustrated by ConBr in Figure 3A, PDB ID: 1AZD, one of our earliest structures). As widely reported, and verified through our extensive studies, the sheets consist of a flat six-stranded one and a curved seven-stranded one, interlinked with loops of variable sizes. Our work, like that of others, also consistently highlighted the crucial dependence on divalent cations (mostly Ca^2+^ and Mn^2+^) for binding capacity. The metal-binding site, proximal to the CRD, is essential for correctly positioning CRD loop residues. An unusual *cis*-peptide bond between an aspartic acid and the preceding residue, stabilized by this metal site, is also critical for orienting the Asp toward the carbohydrate ligand.^52,53^

**Figure 3:**
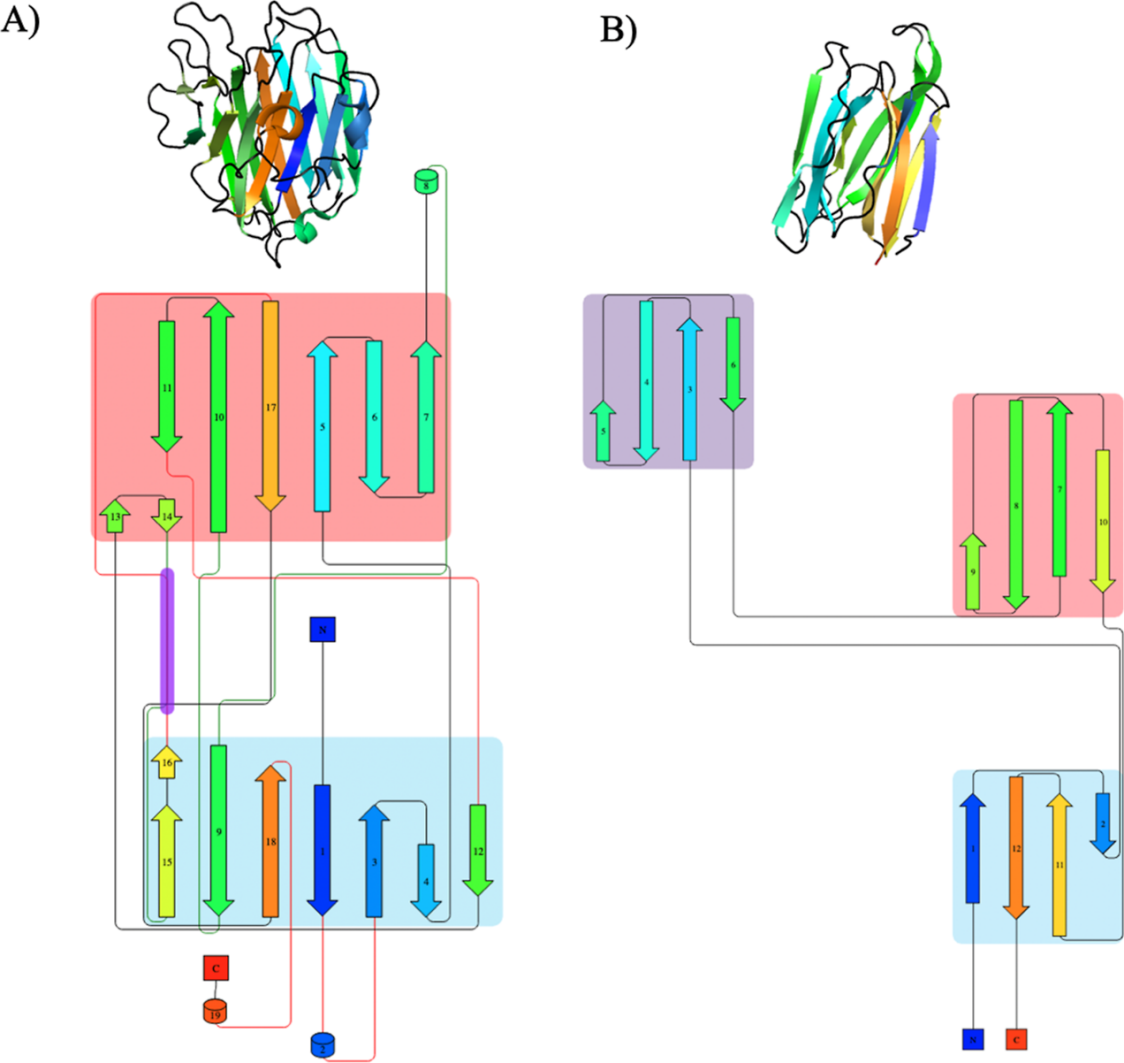
Comparison of the structural organization of legume lectins (A) and Jacalin-related lectins (B). The image depicts ribbon representations of the proteins (top) alongside their corresponding topology diagrams (bottom), highlighting β-strand connectivity and domain architecture. Secondary structure elements are color-coded for clarity.

While foundational studies, such as those by Sharma and Surolia (1997),^54^ had already outlined determinants of specificity, our solving of a diverse array of legume lectin structures at BioMol-Lab provided a rich data set to validate, refine, and observe these principles in action. Indeed, the collective body of structures from BioMol-Lab strongly reinforces the correlation between legume lectin specificity and the length of Loop D within the CRD. Specifically, mannose/glucose-specific lectins we characterized, such as ConBr (Section 2.1), consistently exhibit a shorter Loop D. This creates a shallower binding pocket that favorably accommodates the axial orientation of specific hydroxyl groups in mannose and glucose (notably O4 and O6). Conversely, *N*-acetylgalactosamine/galactose-specific lectins, exemplified by VML from our work in collaboration with the Gruber lab (discussed in Section 2.3), typically possess longer Loop D sequences. This provides the necessary space and additional interaction points (e.g., through backbone amides or side chains like Ser or His) for the different spatial arrangement of hydroxyl groups in galactose and its derivatives (interacting with O3 and O4). While conserved Asp, Gly, and Asn residues in Loops A, B, and C provide the core hydrogen-bonding scaffold, the length and conformation of Loop D effectively “gates” access to these key residues, acting as a primary determinant of monosaccharide preference. This principle also extends to other specificities; GlcNAc and fucose-specific lectins often display intermediate Loop D lengths. For example, while not a primary focus of our lab, the structure of fucose-specific lectins like LTL demonstrates how a unique Trp residue in Loop D provides stacking and hydrogen bonding interactions, compensating for the absence of the O6 hydroxyl in fucose. The sequence variability within Loop D, even for a given length, can further tune specificity. Sharma and Surolia also noted that Glycine and Proline residues within the loops can significantly impact their conformation, introducing sharper turns, a feature consistent with our observations. Moreover, Loop C, despite its high conservation in length within specificity groups, can modulate the binding site’s “openness” and contribute to preferences for specific substituents, as our studies on various Diocleinae lectins have subtly indicated. These structural determinants, repeatedly observed in our two decades of work, offer a robust framework for understanding and predicting legume lectin specificity.

Our extensive work on ConA-like lectins and others (detailed throughout Section 2) also illuminated the remarkable diversity in their quaternary structures, which directly impacts biological function. While legume lectins share a conserved fold, they range from dimers to tetramers, and occasionally higher-order oligomers. We frequently encountered the homodimer, often formed by a “canonical” association where the six-stranded β-sheets of two monomers create an extended 12-stranded β-sheet (as in peanut agglutinin). However, alternative dimerization modes exist (e.g., the “back-to-back” EcorL arrangement or the unique DB58 association). Tetramers, the next most prevalent state, also show diverse arrangements, arising from associated canonical dimers (like the ConA-like tetramers we extensively studied, e.g., ConBr, DGuiL, CFL, Cbol) or entirely different interfaces (e.g., Griffonia simplicifolia lectin I 30). A key phenomenon we investigated in several Diocleinae lectins (see Section 2.1 and 2.2 for ConBr, CFL, Cbol) is the influence of pH on oligomerization.

Beyond our primary focus on ConA-like legume lectins, BioMol-Lab’s exploration of Brazilian biodiversity, as recounted in Section 2.1, led us to broaden our structural analysis to other plant lectin families, revealing further diversity in carbohydrate-binding architectures. Besides legume lectins, we explored both Chitinase-related agglutinins and Jacalin-related lectins, which possess distinct folds compared to the legume lectin jelly roll (Figure 3B). Our work on Parkia platycephala (Section 2.1) proved particularly illuminating, yielding the first crystal structures of lectins from the Mimosoideae subfamily. The initial structure determined was that of P. platycephala seed lectin (PPL), a mannose/glucose-specific lectin (PDB IDs: 1ZGR, 1ZGS). Despite being found in a legume, its main constitutive lectin is from the Jacalin-related family; this lectin revealed a novel dimeric assembly of three tandemly repeated β-prism domains per monomer, forming a toroid-shaped molecule (Figure 1B). This arrangement created six mannose-binding sites positioned around the periphery of the dimer, with differing modes of mannose interaction across the sites, highlighting the structural versatility of the β-prism fold. Subsequently, we characterized and determined the crystal structure of P. platycephala lectin 2 (PPL2), an endochitinase with *N*-acetylglucosamine-binding activity (PDB ID: 2GSJ). PPL2 presented a completely different fold, a (β/α)­8-barrel (TIM-barrel), characteristic of glycosyl hydrolase family 18 chitinases. The PPL2 structure, refined to 1.75 Å resolution, allowed detailed analysis of the catalytic residues and the chitin-binding cleft, confirming its relationship to other chitinases. The presence of these two structurally unrelated lectins within the same plant species highlights the complexity of the plant defense system and its evolutionary adaptations.

The cumulative body of work at BioMol-Lab, detailed through our narrative in Section 2 and synthesized here, has consistently shown that lectins, much like Proteus, are defined by their adaptability and structural plasticity. Even subtle variations, whether in loop length (as discussed with Loop D), quaternary structure (like the pH-dependent changes we observed in CFL and Cbol), or the noncanonical binding sites we discovered (Section 2.2), can profoundly reshape their specificity and biological activities. Beyond their well-known carbohydrate recognition, lectins exhibit unexpected functional diversity, from hormone regulation to plant defense, as seen with the IAA and Abu binding sites investigated by our lab. By accepting this complexity and continually refining our approaches, we have not only deepened our understanding of lectin biology but also uncovered new possibilities for their biotechnological applications. The key lesson from our decades of research is that in the lectin world, even the smallest changes can lead to transformative discoveries.

## 4 Future Horizons in Lectin Research

Despite significant advancements, the field of plant lectinology continues to present intriguing questions and is dynamically evolving with the advent of new technologies. This section outlines some key unanswered questions that drive current research and discusses emerging trends poised to reshape our approach to understanding lectin structure and function.

### 4.1 Unanswered Questions

Even in a field as established as plant lectinology, it is clear that many questions remain unanswered to this day, and pursuing those answers will continue to drive lectin research for years to come. High-resolution techniques like X-ray crystallography, cryo-electron microscopy (cryo-EM), and integrative computational approaches will undoubtedly be at the forefront of these efforts.

One pressing question that we have been trying to pursue over all these years is the relationship between the structural diversity of plant lectins and their functions as well as the reason that lectins can be found in all organisms. It is clear that plant lectins exhibit remarkable variability in structural architecture, ranging from monomers to complex oligomers. While a great deal is known about the structure and binding mechanisms of many plant lectins, the ways in which these diverse structures influence function is still actively being researched. For example, why do lectins from different families exhibit specificity toward the same sugars despite having dramatically different structures? Structural and functional studies will be essential to solving this mystery.

Beyond this overarching question, many others persist. For instance, the molecular basis behind the fine specificity of plant lectins toward different glycans, even when their binding motifs are similar, remains elusive. While hydrogen bonds and CH-π interactions are universally recognized as important contributors to specificity, as seen in cocrystallization studies, less-studied factors such as water-mediated interactions and the conformational flexibility of both lectin residues and glycans may be just as significant.

Extended binding sites and the effects of multivalency in some plant lectins represent another underexplored area. The multivalency property is typically used to offset the relatively low binding affinity of lectins to carbohydrates. These additional binding sites may modulate binding specificity or further enhance multivalency, significantly contributing to their functionality in complex biological systems.^55,56^ Furthermore, hydrophobic subsites within extended binding regions are another intriguing but poorly understood feature that warrants further investigation. Noncanonical subsites could play a role in plant development by holding hormones and other molecules as seen in previous sections. Additionally, oligomerization patterns are critical for function; some lectins form dimers or higher-order oligomers, which can create additional binding surfaces, increasing avidity for carbohydrate ligands.^57^

Additionally, the impact of post-translational modifications on plant lectin structure and function remains speculative. Plant lectins are often subject to complex processing, and modifications like glycosylation could influence their oligomerization states or binding properties in ways that are not yet fully understood. Finally, the integration of computational tools, such as molecular dynamics simulations and machine learning, presents exciting opportunities to model lectin-glycan interactions, predict binding behaviors, and uncover new facets of plant lectin biology. In our view, the integration of computational modeling with experimental approaches holds immense promise in overcoming these barriers.

### 4.2 Emerging Trends

Right now, we are witnessing a significant shift in how structural biology is conducted, with structural studies of plant lectins being, and set to be, profoundly influenced by emerging techniques such as AI-based methods, cryo-electron microscopy (Cryo-EM), and the increasingly advanced capabilities of X-ray crystallography.

Cryo-EM has become a transformative tool in structural biology, offering many opportunities to study complex biological systems with near-atomic resolution. While its application to plant lectins remains limited, the technique has already proven its value in investigating a wide range of challenging macromolecular structures, showcasing its potential for future studies in this area. For example, it has been instrumental in resolving the structures of membrane proteins, which are notoriously difficult to crystallize due to their hydrophobic nature and conformational flexibility. This includes small membrane proteins such as transporters and G-protein-coupled receptors (GPCRs), where the technique has provided insights into their dynamic conformational states and mechanisms of action.^58,59^ Similarly, large protein complexes that are unsuitable for crystallization have been successfully studied using Cryo-EM. Examples include the Type IV secretion system, a multiprotein assembly involved in molecular transport across membranes, and energy-converting complexes like ATP synthase and photosystem I, which are critical for cellular energy production.^60^ Cryo-EM has also revolutionized the field of structural virology by enabling detailed studies of viral particles and their components. A notable example is the SARS-CoV-2 spike protein, where Cryo-EM has revealed its conformational changes during receptor binding and fusion, providing key insights for vaccine development.^61^ Other viruses, such as Zika and HIV, have similarly been analyzed to understand their structure–function relationships and interactions with host factors or therapeutic agents.^62,63^ Beyond these applications, Cryo-EM has demonstrated its capability to study transient and flexible protein–ligand interactions. For instance, it has been used to visualize ribosome-tRNA complexes and other dynamic assemblies, shedding light on molecular mechanisms that were previously inaccessible.^64^ Although plant lectins have not yet been widely studied using Cryo-EM, the technique’s ability to resolve multimeric and dynamic proteins suggests it could be highly effective for this purpose. The ability to capture lectin-carbohydrate complexes in near-native states would allow researchers to explore binding interactions and conformational changes.

Despite the impressive advancements in Cryo-EM, we are confident that X-ray crystallography will remain a cornerstone of structural biology, largely due to the relatively lower costs and reduced computational demands for data processing. Moreover, the evolution of synchrotron sources, such as the Brazilian Sirius synchrotron with its MANACÁ beamline, provides powerful tools for lectin researchers. The MANACÁ beamline, a fourth-generation macromolecular crystallography facility, is optimized for high flux and offers advanced methodologies like room-temperature and serial crystallography. Its capabilities include automated sample handling, rapid data collection, and cutting-edge microfluidic devices for fixed-target and in-flow delivery systems, making it ideal for studying intricate molecular interactions.^65^ High-resolution structures, now routinely achievable with facilities like MANACÁ, allow us to explore detailed carbohydrate-binding domains and water-mediated interactions, which are often critical for binding specificity and functionality.

Another breakthrough we are observing is the advent of deep learning models, particularly AlphaFold3, which has revolutionized protein structure prediction and modeling, including that of plant lectins. AlphaFold3 applies evolutionary information and advanced neural network algorithms to predict lectin structures and their interactions with remarkable accuracy.^66^ This will prove important for lectins with unresolved experimental structures or complex carbohydrate interactions. Additionally, these predictive models can guide experimental studies by identifying potential binding sites and interaction regions. When combined with structural data from Cryo-EM and X-ray crystallography, AlphaFold3 enhances our ability to design lectins with specific binding properties for various biotechnological applications. Although AlphaFold’s usage in lectin research is currently limited, We anticipate significant growth in its application. Its impressive reliability has the potential to start a major transformation in the field. We are actively contributing to the experimental data landscape that supports and complements such predictive approaches, as exemplified by the chronological deposition of diverse lectin structures by BioMol-Lab (Figure 4), which enrich the structural databases utilized by these AI tools.

**Figure 4:**
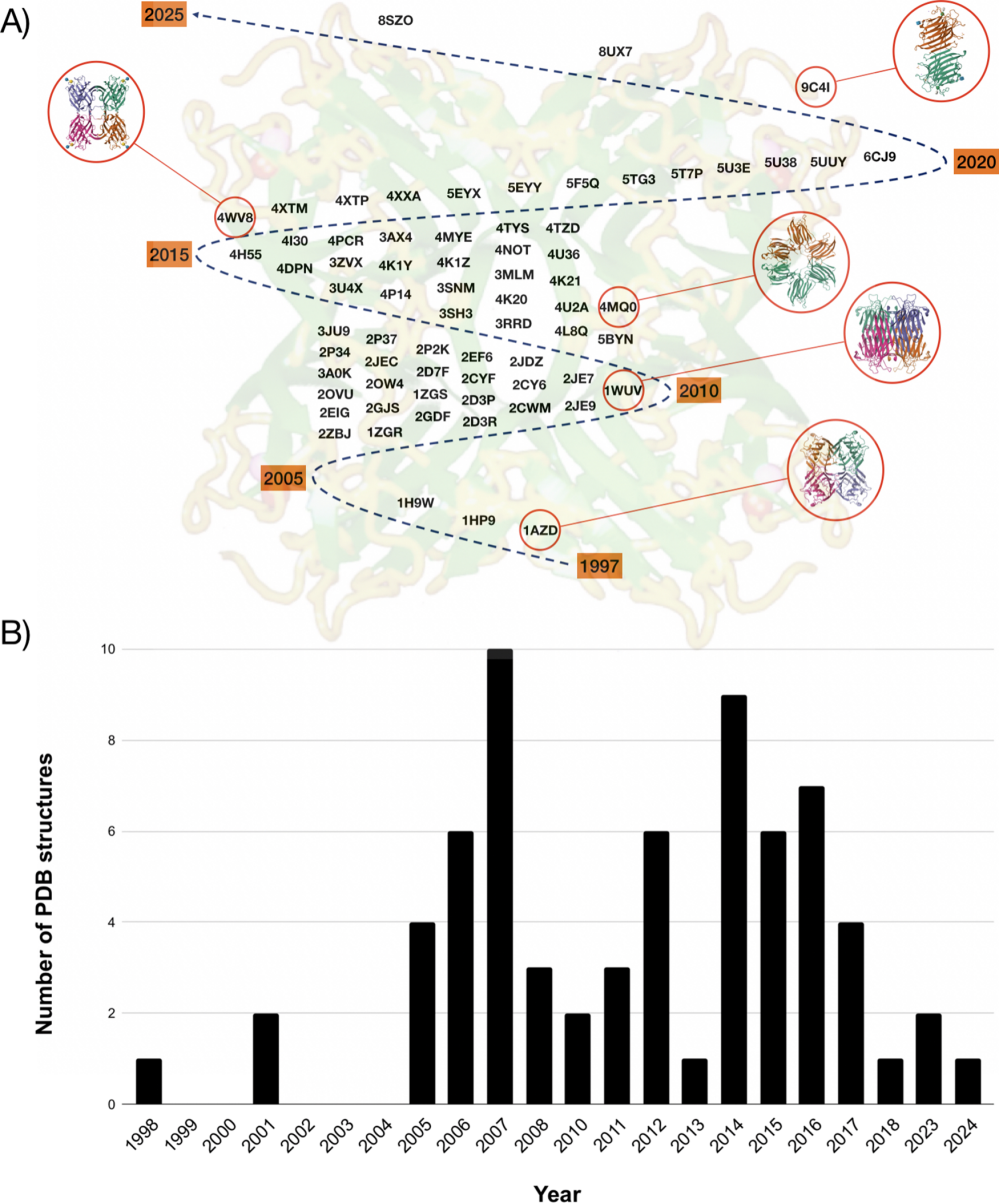
Structures solved by BioMol Lab. (A) Timeline of deposited structures, grouped into chronological ranges with representative examples. (B) Number of PDB structures deposited per year.

This forward-looking perspective highlights that while many fundamental questions about lectin specificity, function, and regulation remain, the synergistic application of advanced experimental techniques, coupled with the predictive power of AI, promises an exciting new era of discovery in lectin science.

## 5 Conclusions

The two-decade journey of lectin crystallography at BioMol-Lab has been sustained by scientific curiosity, strategic collaborations, and a commitment to learning. Starting from a position of heavy reliance on international expertise, we evolved into an independent structural biology center, pioneering protein crystallography in Northeast Brazil and making significant contributions to the understanding of legume lectin structure–function relationships. Our work has consistently demonstrated that seemingly minor structural variations can profoundly impact lectin specificity, binding affinity, and biological activity. The discovery of conserved, yet functionally diverse, binding sites beyond the canonical carbohydrate-recognition domain underscores the complexities and the necessity of more studies within lectinology.

The integration of X-ray crystallography and computational methods has propelled our research beyond static structural descriptions, allowing us to probe the dynamic nature of lectin-ligand interactions and to predict functional consequences. The collaborative spirit, both within Brazil (e.g., with LNLS/Sirius) and internationally, remains crucial for accessing cutting-edge technologies and expertise.

Looking ahead, the field of lectin structural biology is poised for continued growth and innovation. The rise of Cryo-EM and AI-driven structural prediction (e.g., AlphaFold) offer exciting new possibilities for tackling challenging structural problems, including membrane-bound lectins and complex lectin-glycan interactions. However, X-ray crystallography, particularly with the advancements in synchrotron facilities like Brazil’s Sirius, will remain a vital tool for high-resolution structural analysis. All of these will help with unanswered questions, particularly concerning the precise molecular mechanisms behind the fine specificity of lectins, the role of extended binding sites and multivalency, and the impact of post-translational modifications. By embracing these emerging trends and continuing to explore the rich biodiversity of Brazil, BioMol-Lab is well-positioned to contribute to the next chapter of lectin research and build its way to a conceptual Proteus 3.0.

## Data Availability

The data supporting the findings of this study are available in the manuscript and the references therein.
